# Effects of different sodium salts and nitrogen sources on the production of 3-hydroxybutyrate and polyhydroxybutyrate by *Burkholderia cepacia*

**DOI:** 10.1186/s40643-021-00418-x

**Published:** 2021-07-19

**Authors:** Jianfei Wang, Jiaqi Huang, Huanyu Guo, Shaoming Jiang, Jinyue Qiao, Xingyu Chen, Zixuan Qu, Wanyue Cui, Shijie Liu

**Affiliations:** 1 Department of Chemical Engineering, SUNY College of Environmental Science and Forestry, Syracuse, NY 13210 USA; 2 The Center for Biotechnology & Interdisciplinary Studies (CBIS), Rensselaer Polytechnic Institute, Troy, NY 12180 USA; 3 School of Engineering, Tufts University, Medford, MA 02155 USA

**Keywords:** 3-Hydroxybutyrate, Polyhydroxybutyrate, *Burkholderia cepacia*, Sodium salts, Nitrogen source

## Abstract

The effects of NaCl, Na_2_SO_4_, Na_2_HPO_4_, and Na_3_C_6_H_5_O_7_ on the production of 3-hydroxybutyrate, polyhydroxybutyrate, and by-products by *Burkholderia cepacia*. Proper addition of Na_3_C_6_H_5_O_7_ can significantly promote the production of 3-hydroxybutyric acid and polyhydroxybutyrate. The concentration, productivity, and yield of 3-hydroxybutyrate were increased by 48.2%, 55.6%, and 48.3% at 16 mM Na_3_C_6_H_5_O_7_. The increases of 80.1%, 47.1%, and 80.0% in the concentration, productivity, and yield of polyhydroxybutyrate were observed at 12 mM Na_3_C_6_H_5_O_7_. Na_2_SO_4_ and Na_2_HPO_4_ also have positive effects on the production capacity of 3-hydroxybutyrate and polyhydroxybutyrate within a certain range of concentration. NaCl is not conducive to the improvement of fermentation efficiency. Compared with a single nitrogen source, a mixed nitrogen source is more conducive to enhancing the production of 3-hydroxybutyrate and polyhydroxybutyrate.

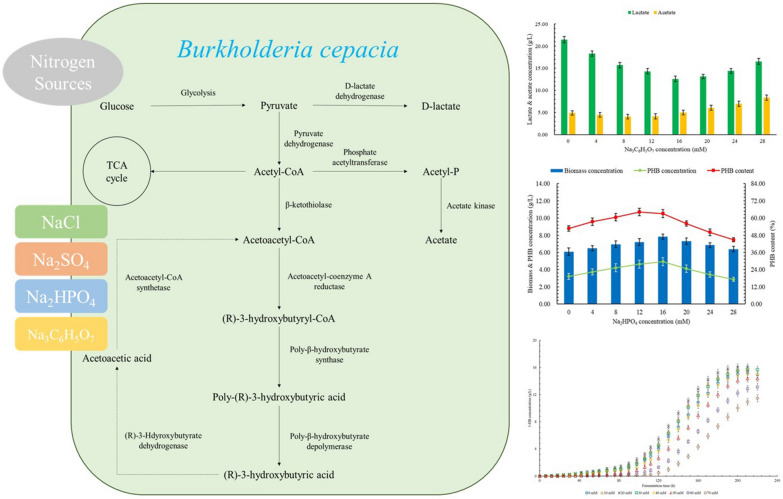

## Introduction

3-Hydroxybutyrate (3-HB) is an organic monomer with both hydroxyl and carboxyl groups, which is considered an important chemical precursor or intermediate to synthesize other monomers, copolymers, and homopolymers with good performance in the chemical industry (Wang and Liu 2014; Anjum et al. 2016). In addition, 3-HB can be widely used in the chemical industry and pharmaceutical industry due to its unique chiral structure as an important precursor to produce antibiotics, vitamins, and other related products (Ren et al. 2010). Polyhydroxybutyrate (PHB) is a kind of polyhydroxyalkanoate (PHA) with 3-HB as the monomer produced during the secondary metabolism of some microorganisms, which has been confirmed to exist in these microorganisms as an important energy storage substance (Koller 2018; Kessler and Witholt 2001). Compared with petroleum-based polymers, PHB has biodegradability and unique mechanical properties, which can be used as an effective substitute for traditional plastics (Aramvash et al. 2018). In the pharmaceutical industry, PHB has become an advanced high-performance medical material due to its good chemical properties and biocompatibility (Koller 2018). The production of various PHAs was estimated to reach $57,000,000 in 2019, and the total market size of PHAs was predicted to reach $98, 000,000 by 2024 (Sirohi et al. 2021).

The studies on the 3-HB usually focus on different production methods, mainly including chemical synthesis, chemical or enzymatic depolymerization of PHB, and in vivo depolymerization of PHB. The chemical synthesis requires a higher level of substrate purity and catalyst performance, which results in increased production costs. Chemical or enzymatic depolymerization of PHB usually requires strict control of process conditions and has a relatively low yield (Wang and Liu 2014). The in vivo depolymerization of PHB can be carried out under mild conditions with a higher yield, and its production efficiency can also be improved more easily by optimizing fermentation conditions and medium components. Therefore, this method has a great development potential, which is of great significance for reducing production costs and technical difficulties. Currently, the biosynthesis of PHB has become a research hotspot. The strains used for PHB synthesis are mainly *Bacillus* (Bomrungnok et al. 2020), *Cupriavidus* (Leong et al. 2019), halophilic archaeal strains (Salgaonkar and Bragança 2017), *Pseudomonas* (Pereira et al. 2021), engineered *Saccharomyces cerevisiae* (Kocharin and Nielsen 2013), and engineered *Escherichia coli* (Bhatia et al. 2015). In recent years, *Burkholderia* species have also been used for PHB production, but the related research is limited.

*Burkholderia cepacia* (*B. cepacia*) is a strain that synthesizes PHB and depolymerizes PHB into (R)-3-HB simultaneously from various carbon sources, which has high production efficiency, stable yield, and high tolerance to various production inhibitors (Dietrich et al. 2013). The theoretical total yield of 3-HB is 0.770 g/g glucose based on Eq. (1):

1 $$3{\text{C}}_{6} {\text{H}}_{6} {\text{O}}_{6} \to 4{\text{CH}}_{3} {\text{CH}}\left( {{\text{OH}}} \right){\text{CH}}_{2} {\text{COOH}} + 2{\text{CO}}_{2} + 2{\text{H}}_{2} {\text{O}}$$

Compared to that of polylactic acid (PLA), the production methods of PHB are significantly different. The PLA is produced by the chemical polymerization of the lactic acid monomer, which is usually obtained by the fermentation process (Wang et al. 2020). However, the PHB is obtained directly from the fermentation process, which is synthesized and accumulated by the metabolism of *B. cepacia* cells. The 3-HB will also be obtained by the in vivo depolymerization of PHB by further metabolism of *B. cepacia*. Therefore, changes in fermentation conditions will simultaneously affect the production rate and yield of PHB and 3-HB.

The studies on the biosynthesis of 3-HB and PHB mainly focus on the optimization of fermentation process parameters (Mohd Zain et al. 2021) and the application of cheap carbon sources (Bhatia et al. 2015). In terms of media component analysis and optimization, current research mainly involves the effects of nitrogen source type and C/N ratio on the synthesis of 3-HB and PHB (Li et al. 2019). However, other related studies, especially the studies on 3-HB production, are still limited. It has been reported that PHB synthesis and accumulation in cells can be achieved by limiting one or more key nutrients, mainly provided by the anions of the inorganic salt, but controlling these anions at a small amount can promote the synthesis and accumulation of PHB or promote the depolymerization of PHB into 3-HB (Wang and Liu 2014). In addition, some anions can also promote the production of PHB and 3-HB by inhibiting cell growth or other metabolic pathways (Azizi et al. 2017). Therefore, the analysis of anion effects and the optimization of anion types and concentrations will have great significance to the production of 3-HB and PHB. The effects of inorganic salts on the extracellular PHB degradation by PHB depolymerase have been reported (Blevins et al. 2018). However, the effect of inorganic salts on the intracellular production of 3-HB and PHB is still rarely studied. In addition, the effects of different nitrogen sources on the production of 3-HB and PHB still need to be further analyzed.

In this study, four types of sodium salts were applied to the production of 3-HB and PHB by *B. cepacia* cells through the batch fermentation process. The effects of each type of sodium salt on the production of 3-HB, PHB, and by-products were studied at eight levels. The effects of different nitrogen sources on the production of the mentioned products were also studied.

## Materials and methods

### Seed culture preparation

The *B. cepacia* ATCC 17759 provided by American Type Culture Collection (ATCC) was stored as freeze-dried cells in a − 80 °C freezer. Before using for PHB and 3-HB production, the *B. cepacia* cells were activated in the seed culture medium for 48 h on a shaker at 220 rpm and 30 °C. The seed culture medium was composed of 5.0 g/L peptone and 5.0 g/L beef extract.

### Batch fermentation

The batch fermentation was conducted in 1.0-L New Brunswick bioreactors with a working volume of 800 ml. The carbon source was 54 g/L glucose. The nitrogen sources were 1 g/L peptone. The environment of key ions for PHB synthesis was created by 20 mg/L CaCl_2_, 80 mg/L MgSO_4_, 50 mg/L ZnSO_4_, 30 mg/L (NH_4_)_5_[Fe(C_6_H_4_O_7_)_2_], and 1 g/L K_2_HPO_4_. The composition was modified based on the study of Wang and Liu (2014) and Rodrigues et al. (2019) to adapt to the different substrate and nitrogen source. The sodium salts with different anions at a specific concentration were added to the fermentation medium before fermentation to study the effects of the type and initial concentration of sodium salts on PHB and 3-HB production by *B. cepacia* cells (Table 1). The pH was automatically controlled at 7.0 by adding 5 M NaOH solution. The temperature was maintained at 28 °C by heat jacket. The aeration and stirring speed were maintained at 50 ml/min and 200 rpm, respectively. The fermentation period was determined based on the time when the concentration of 3-HB reached the highest concentration. The effects of different nitrogen sources on the synthesis of various products were studied with single nitrogen sources and mixed nitrogen sources. The single nitrogen sources included peptone, yeast extract, urea, and (NH_4_)_2_SO_4_. The mixed nitrogen sources were composed of two single nitrogen sources with a weight ratio of 1:1. All single nitrogen sources and mixed nitrogen sources were maintained at 1 g/L, and other fermentation conditions remained the same.

**Table 1:** The type and concentration level of sodium salts

Sodium salt	Concentration level (mM)							
	1 (Control)	2	3	4	5	6	7	8
NaCl	0	10	20	30	40	50	60	70
Na_2_SO_4_	0	4	8	12	16	20	24	28
Na_2_HPO_4_	0	4	8	12	16	20	24	28
Na_3_C_6_H_5_O_7_	0	4	8	12	16	20	24	28

### Determination of cell biomass concentration

The concentration of cell biomass was calculated based on the optical density (OD) at a wavelength of 600 nm measured by an OD scanner (BugLab, CA, USA). A calibration curve showing the linear relationship between concentration of cell biomass and OD value was applied.

### Determination of 3-HB, lactate, and acetate

The concentrations of 3-HB, lactate, and acetate were determined by 1H nuclear magnetic resonance (NMR) spectroscopy based on the method provided by Wang and Liu (2014). The NMR samples were composed of 0.1 ml internal standard, 0.4 ml D_2_O, and 0.5 ml fermentation broth sample. The internal standard solution was prepared by dissolving 4.2% (w/w) glucosamine, 0.2% (w/w) trimethylamine, and 0.1% (w/w) trimethylsilyl propionate, and it was stored in − 10 °C freezer after preparation. The product concentration was calculated with a linear calibration curve between the mass concentration and the area of the corresponding peak shown on the NMR spectrum.

### Determination of PHB

The harvested cell biomass was centrifuged at 4000 × g for 10 min and subsequently freeze-dried. The freeze-dried cells were subsequently dispersed by the mixture solution consisting of 2 ml sodium hypochlorite and 2 ml chloroform at 60 °C for 24 h. The PHB dissolved in the organic phase was obtained by separating the aqueous phase with cell debris after centrifugation at 4000 × g for 20 min. The PHB solution was poured into a 10 cm glass petri dish and subsequently placed in a fume hood to evaporate the organic solvent for 10 h to recycle the PHB film. Samples for 1H NMR quantification were prepared and analyzed based on the method provided by Jan et al. (1996). Samples were prepared by dissolving the PHB film into 1 ml CDCl_3_. The 1% methanol and 1 ‰ benzene were also added to the sample solution as the internal standard.

### Statistical analysis

The experimental data were shown as “mean value ± standard deviation” based on triplicate runs of the experiment. The data were analyzed based on a T-test by Minitab 18 with a 95% level of significance.

## Results and discussion

### Effects of NaCl on the production of 3-HB, PHB, and by-products

**Fig. 1:**
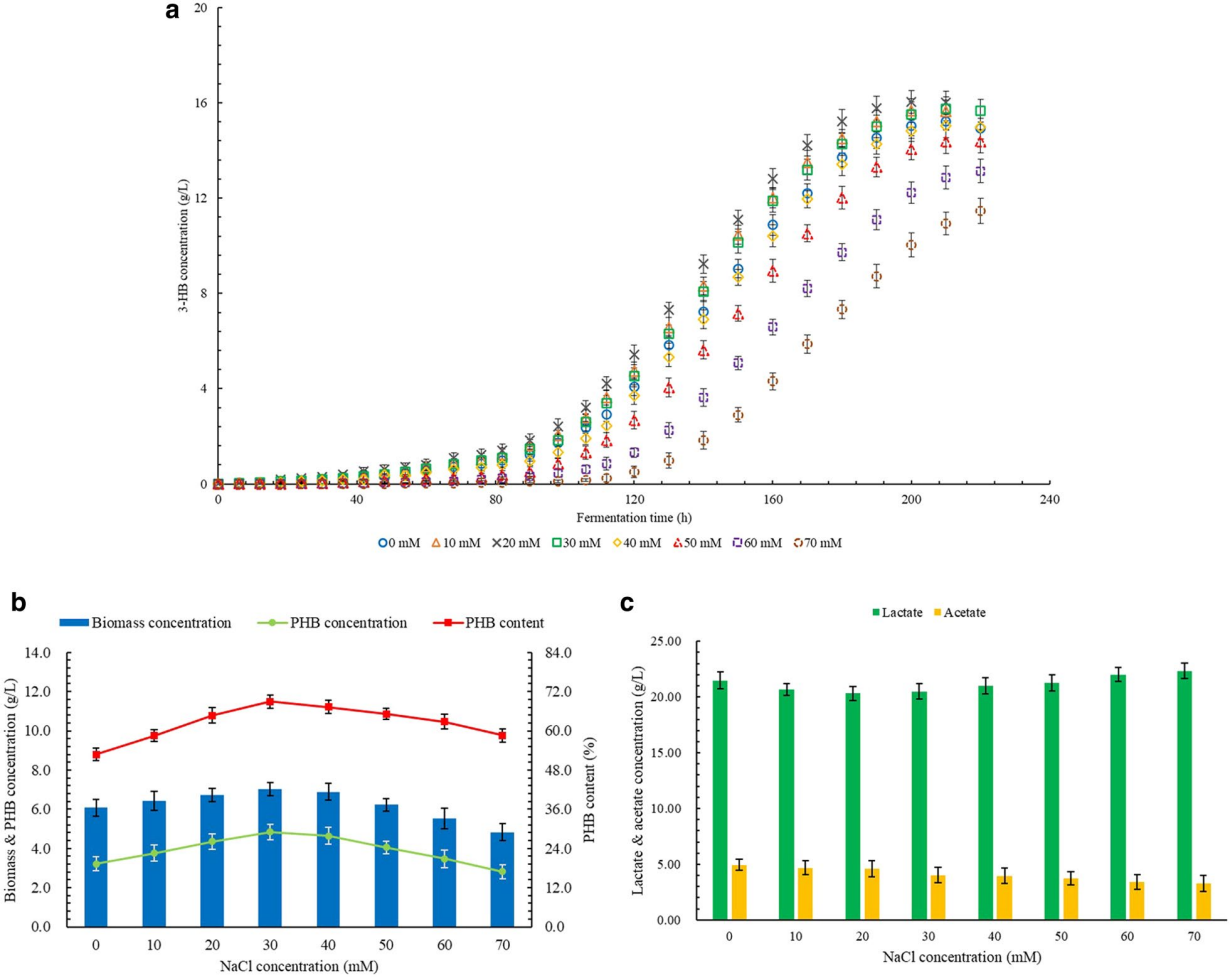
**a** Effects of NaCl on the production of 3-HB. (**b**) Effects of NaCl on the production of PHB. (**c**) Effects of NaCl on the production of LA and AC

The results of 3-HB production at different levels of NaCl concentration are shown in Fig. 1a. When there was no variable salt added, the concentration of 3-HB obtained from batch fermentation was 15.22 g/L. The concentration of 3-HB did not show significant difference when the NaCl concentration was between 0 and 40 mM. However, when NaCl concentration reached 50 mM, the 3-HB concentration was 5.5% lower than the control group. The final 3-HB concentration of 11.45 g/L was obtained at NaCl concentration of 70 mM, which was 24.8% lower than the control group.

The effects of different NaCl concentration levels on the PHB production are shown in Fig. 1b. The PHB concentration of 3.21 g/L with a PHB content of 52.8% was obtained with the control group. The highest concentration and content were obtained at NaCl concentration of 30 mM as 4.84 g/L and 68.9%, respectively, which were 50.8% and 30.5% higher than the control group, respectively. At NaCl concentration of 60 mM, the PHB concentration was not significantly different from the control group, but the PHB content was significantly higher. The PHB content of 58.6% at 70 mM NaCl was still 11.0% higher than the control group with insignificant difference of PHB concentration.

Lactate (LA) and acetate (AC) were regarded as by-products in the production of 3-HB and PHB by *B. cepacia*. The LA and AC concentrations of the control group were 21.48 g/L and 4.94 g/L, respectively. As shown in Fig. 1c, the LA concentration did not change significantly in the range of NaCl concentration. The AC concentration showed a downward trend with the increase in NaCl concentration. The lowest AC concentration was obtained as 3.27 g/L at NaCl concentration of 70 mM, which was 33.8% lower than the control group.

Table 2 shows the yield and productivity of 3-HB and PHB at different levels of NaCl concentration. The highest productivity of 3-HB was obtained at 20 mM NaCl, which was 10.6% higher than the control group. However, the highest yield of 3-HB was obtained at 30 mM NaCl, which was 4.1% higher than the control group. Both highest productivity and yield of PHB were obtained at 30 mM NaCl, which were 41.8% and 33.6% higher than the control group, respectively.

**Table 2:** The productivity and yield of 3-HB and PHB at different NaCl concentrations

Concentration, mM	Productivity, g/(L × h)		Yield, g/g glucose	
	3-HB	PHB	3-HB	PHB
0	0.073 ± 0.002	0.015 ± 0.002	0.298 ± 0.005	0.063 ± 0.006
10	0.078 ± 0.003	0.019 ± 0.002	0.303 ± 0.007	0.074 ± 0.007
20	0.080 ± 0.002	0.022 ± 0.002	0.307 ± 0.006	0.085 ± 0.007
30	0.075 ± 0.002	0.023 ± 0.002	0.311 ± 0.007	0.095 ± 0.007
40	0.072 ± 0.002	0.022 ± 0.002	0.297 ± 0.007	0.092 ± 0.008
50	0.065 ± 0.002	0.018 ± 0.001	0.289 ± 0.007	0.080 ± 0.006
60	0.060 ± 0.002	0.016 ± 0.002	0.258 ± 0.007	0.068 ± 0.008
70	0.052 ± 0.002	0.013 ± 0.002	0.225 ± 0.008	0.056 ± 0.007

Some studies have reported that the proper concentration of NaCl will promote biosynthesis of PHB (Thapa et al. 2018; Azizi et al. 2017). The effects of NaCl at different concentrations on cell growth and metabolism were mainly related to the change in osmotic pressure. Appropriate addition of NaCl can improve the osmotic pressure environment and increase the overall metabolic efficiency of cells, thereby simultaneously promoting the production of PHB and 3-HB (Wood 2015). It has also been pointed out that the promotion of the decomposition of PHB to produce 3-HB may be due to greater energy demand to cope with changes in osmotic pressure (Rodríguez-Contreras et al. 2016). With the increase in the NaCl concentration, the osmotic pressure increases, which leads to the dehydration of cells. However, dehydration provides more space for the accumulation of PHB. Therefore, the efficient synthesis and accumulation of PHB at relatively higher NaCl concentrations is regarded as the process to replace water to maintain the osmotic pressure of cells (Azizi et al. 2017).

When the NaCl concentration is further increased, a higher degree of cell dehydration occurs, and the inhibitory effect of chloride ions on cell growth and PHB synthesis begins to manifest, resulting in reduced levels of cell growth, PHB synthesis, and 3-HB production. However, due to the requirement to maintain the osmotic pressure of cells, the accumulation level of PHB under high NaCl concentration is still high. It explains why the PHB content of cells at 70 mM NaCl is still higher than that of the control group. Under the appropriate NaCl concentration, the metabolic pathway of PHB synthesis and decomposition are more active, so more substrates are converted into intermediate products, such as pyruvate and acetyl-CoA, and subsequently flow to the metabolic pathway of PHB and 3-HB production, rather than the synthesis of LA and AC. As the osmotic pressure rises due to the further increase of the NaCl concentration, although the PHB content is still maintained at a high level, the PHB concentration and synthesis efficiency decrease significantly, which also leads to a reduction in the yield and productivity of 3-HB. In addition, due to more energy demand to cope with the negative effects of high osmotic pressure on cells, more 3-HB is reused, thereby resulting in a reduction in concentration and yield of 3-HB. However, due to the decline in the overall cell metabolism, the level of LA production did not increase significantly. The decrease in AC concentration might be due to the preferential flow of substrate to other pathways under the increased concentration of NaCl.

### Effects of Na_**2**_SO_**4**_ on the production of 3-HB, PHB, and by-products

**Fig. 2:**
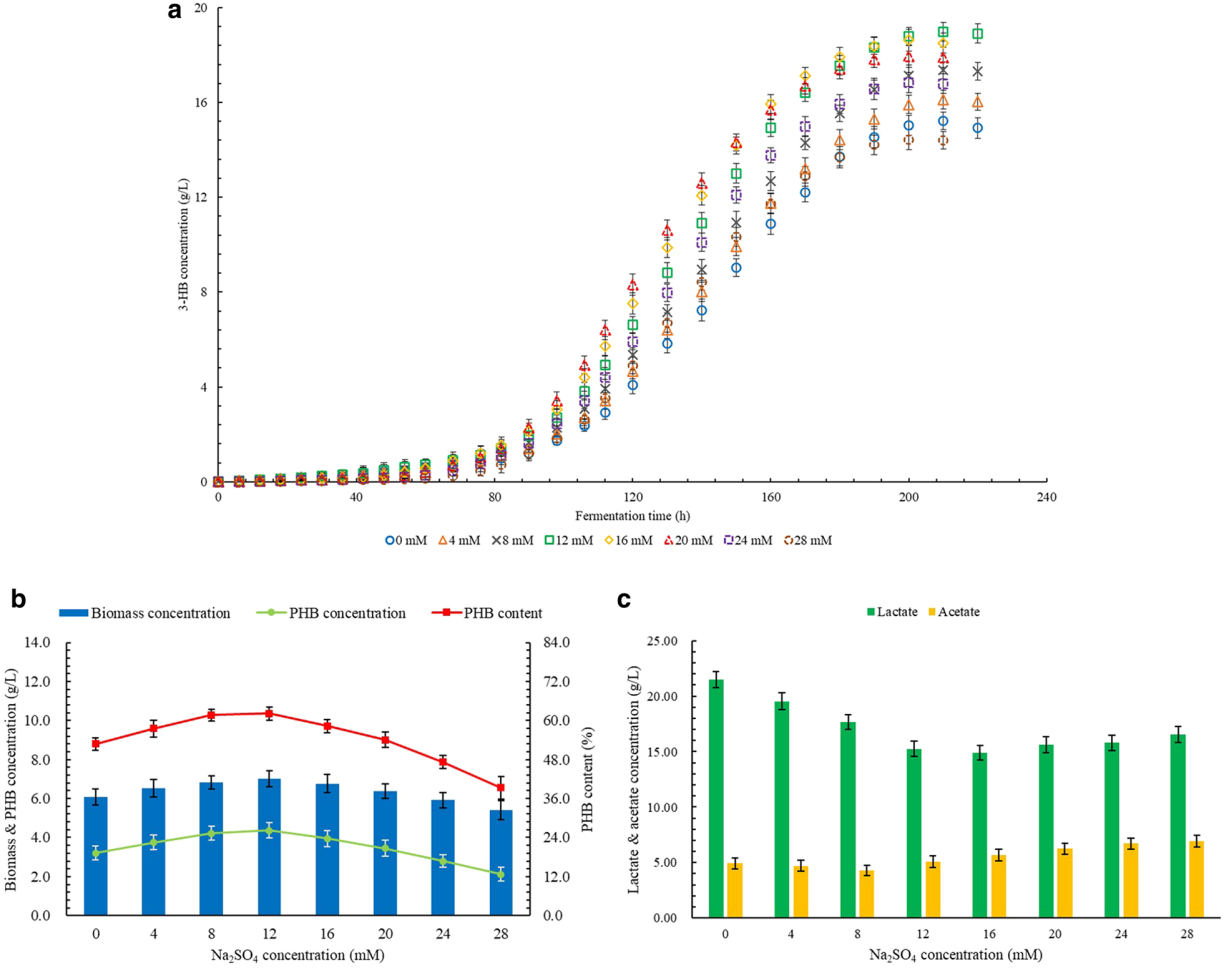
**a** Effects of Na2SO4 on the production of 3-HB. **b** Effects of Na2SO4 on the production of PHB. **c** Effects of Na2SO4 on the production of LA and AC

The effect of different Na_2_SO_4_ concentrations on 3-HB production is shown in Fig. 2a. With the increase in Na_2_SO_4_ concentration from 0 to 12 mM, the final 3-HB concentration increased and reached 18.98 g/L as the highest value, which was 24.7% higher than the control group. When the Na_2_SO_4_ concentration reached 24 mM, the concentration and of 3-HB was 11.3% lower than the highest value but still 17.6% higher than the control group. The lowest final 3-HB concentration of 14.42 g/L was obtained at 28 mM Na_2_SO_4_, which was 5.3% lower than the control group.

As shown in Fig. 2b, both concentration and content of PHB show a parabolic trend with the changes in Na_2_SO_4_ concentration. Both highest PHB concentration of 4.36 g/L and highest PHB content of 62.1% were obtained at 12 mM Na_2_SO_4_, which were 35.8% and 17.6% than the control group, respectively. However, the differences in the concentration and content of PHB between 8 and 12 mM Na_2_SO_4_ were not significantly different. As the further increase in Na_2_SO_4_ concentration, the concentration and content of PHB decreased fast. When the Na_2_SO_4_ concentration reached 24 mM, the PHB content was significantly lower than the control group, but the PHB concentration did not show a significant difference. The lowest concentration and content of PHB were obtained at 28 mM Na_2_SO_4_ as 5.4 g/L and 39.3 g/L, which were 11.2% and 34.4% lower than the control group.

The effects of Na_2_SO_4_ concentration on the concentrations of by-products are shown in Fig. 2c. As the Na_2_SO_4_ concentration increased from 0 to 16 mM, the LA concentration decreased fast, and the lowest LA concentration was obtained as 14.91 g/L, which was 30.6% lower than the control group. With the further increase in Na_2_SO_4_ concentration, the LA concentration increased gradually, but the LA concentration of 16.54 g/L was still 23.0% lower than the control group. The AC concentrations did not significantly change between Na_2_SO_4_ concentrations of 0 to 16 mM. However, the AC concentration further increased with the increase in Na_2_SO_4_ concentration, and the highest AC concentration of 6.94 g/L was obtained at 28 mM Na_2_SO_4_, which was 40.5% higher than the control group.

As shown in Table 3, the highest 3-HB productivity was obtained at 16 mM Na_2_SO_4_, which was 28.4% higher than the control group. The highest 3-HB yield was obtained at 12 mM Na_2_SO_4_, which was 24.7% higher than the control group. Both the highest productivity and yield of PHB were obtained at 12 mM Na_2_SO_4_, and both were 35.9% higher than the control group.

**Table 3:** The productivity and yield of 3-HB and PHB at different Na_2_SO_4_ concentrations

Concentration, mM	Productivity, g/(L × h)		Yield, g/g glucose	
	3-HB	PHB	3-HB	PHB
0	0.073 ± 0.002	0.015 ± 0.002	0.298 ± 0.005	0.063 ± 0.006
4	0.077 ± 0.002	0.018 ± 0.002	0.316 ± 0.005	0.074 ± 0.007
8	0.083 ± 0.002	0.020 ± 0.002	0.340 ± 0.005	0.083 ± 0.006
12	0.091 ± 0.002	0.021 ± 0.002	0.372 ± 0.005	0.086 ± 0.007
16	0.093 ± 0.002	0.020 ± 0.002	0.365 ± 0.005	0.077 ± 0.008
20	0.090 ± 0.002	0.017 ± 0.002	0.352 ± 0.006	0.068 ± 0.007
24	0.084 ± 0.002	0.014 ± 0.003	0.330 ± 0.006	0.055 ± 0.006
28	0.072 ± 0.002	0.011 ± 0.002	0.283 ± 0.006	0.042 ± 0.007

At present, studies on the production of PHB and 3-HB with Na_2_SO_4_ is still limited. However, the positive effect of Na_2_SO_4_ on PHB synthesis has been confirmed (Ramezani et al. 2015). For the growth and metabolism of cells, SO_4_^2−^ is usually used as a nutrient ion to provide the necessary sulfur for the synthesis of some essential amino acids (Yadav et al. 2017). Therefore, SO_4_^2−^ is a key nutritional factor that promotes cell growth and reproduction. Appropriate addition of Na_2_SO_4_ is beneficial to increase the overall metabolic rate of cells, which promotes both synthesis and degradation efficiency of PHB, thereby leading to simultaneous increases in the concentration, yield, and productivity of 3-HB and PHB. This might be due to the participation of SO_4_^2−^ in the synthesis of amino acids for CoA-SH production. CoA-SH is further converted into acetyl-CoA rapidly under a high C/N ratio, which improves the efficiency of related redox reactions in cells, thereby increasing the production rate of intermediate or final products in various metabolic pathways (Doi et al. 1989). Under the effects of a relatively lower concentration of Na_2_SO_4_, more pyruvate is converted into acetyl-CoA, and subsequently flows to the PHB synthesis pathway and the tricarboxylic acid (TCA) cycle instead of the LA synthesis pathway, which leads to a decrease in LA concentration. The decrease in AC concentration may also be due to more acetyl-CoA flowing into other metabolic pathways. The higher overall metabolism of cells also leads to a higher cell growth rate, so the higher cell density is also an important reason for higher overall PHB accumulation and 3-HB production. In addition, a proper concentration of SO_4_^2−^ can promote the uptake of nitrogen sources by cells, and some amino acids can be broken down into acetyl-CoA as the precursor of PHB (Uchino et al. 2007). It may also lead to increased production of PHB and 3-HB. With the further increase of Na_2_SO_4_ concentration, cell growth is significantly promoted, which leads to the difficulty of PHB production and accumulation due to the negative effect of a higher level of CoA-SH (Alcântara et al. 2020). In the early stage of fermentation, the metabolic pathways related to cell growth become more active, which leads to the weakening of the PHB synthesis pathway. This may be the reason for the lower 3-HB concentration at a higher concentration in the early fermentation stage. In the middle stage of fermentation, the synthesis and decomposition of PHB became the main process, which may be because the cell growth rate decreased significantly due to the consumption of nitrogen sources. However, the presence of SO_4_^2−^ still promotes the decomposition of PHB, resulting in a significant increase in the 3-HB production at a higher cell density. The increase in LA concentration is also caused by higher cell density. The increase in AC concentration might be because 3-HB is consumed as a substrate to regenerate acetyl-CoA and flows to the AC synthesis pathway subsequently, which is also related to the higher level of CoA-SH synthesis. Although the production efficiency of 3-HB has been significantly improved in the middle stage of fermentation under a higher Na_2_SO_4_ concentration, the concentration of 3-HB is still lower than that under lower levels of Na_2_SO_4_ concentration, which is mainly caused by the consumption of more glucose and even 3-HB by other metabolic pathways.

### Effects of Na_**2**_HPO_**4**_ on the production of 3-HB, PHB, and by-products

**Fig. 3:**
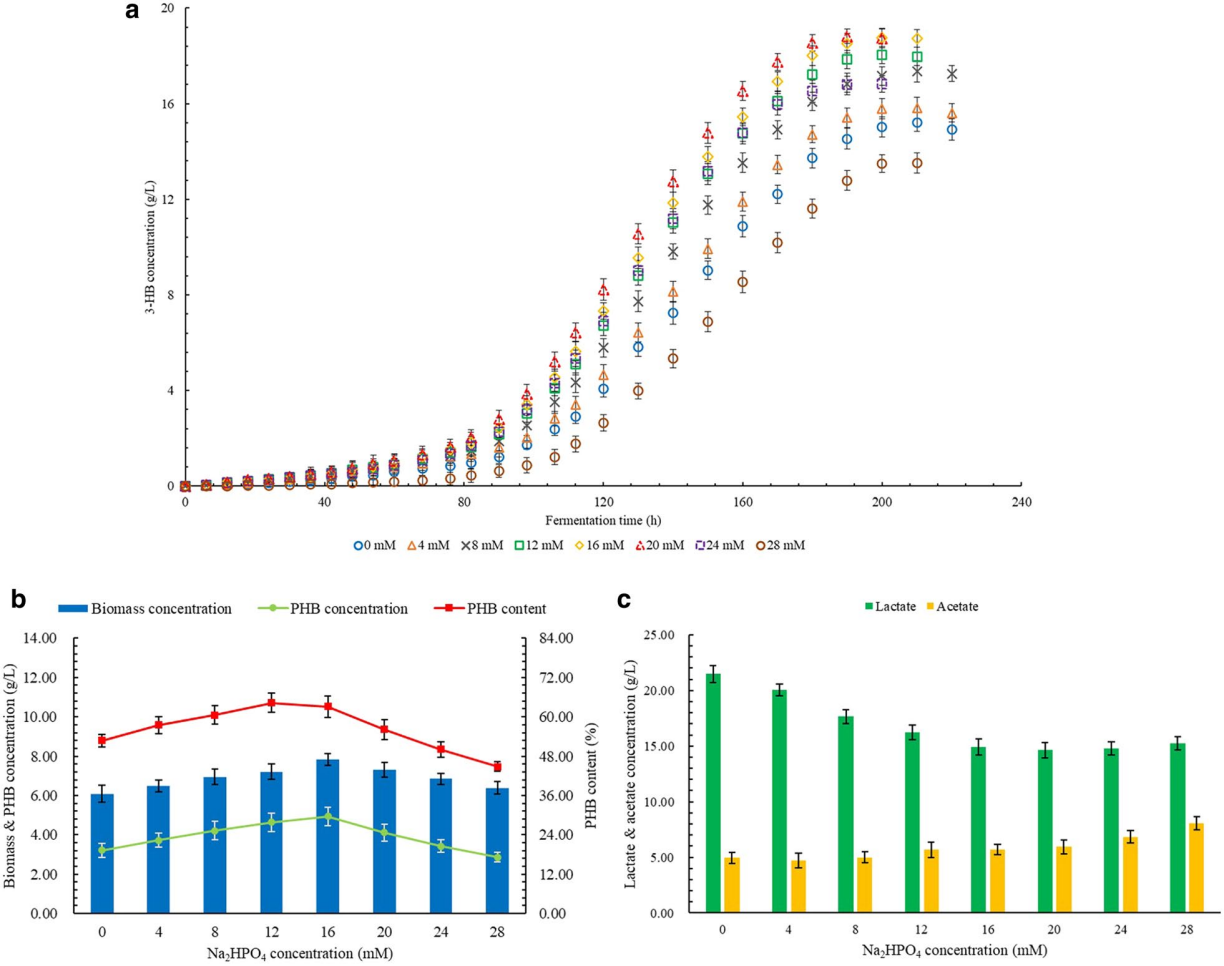
**a** Effects of Na2HPO4 on the production of 3-HB. **b** Effects of Na2HPO4 on the production of PHB. **c** Effects of Na2HPO4 on the production of LA and AC

The results of 3-HB production affected by the different Na_2_HPO_4_ concentrations are shown in Fig. 3a. The increased concentration of 3-HB was observed with the increased Na_2_HPO_4_ concentration from 0 to 20 mM. The highest final concentration of 3-HB was obtained as 18.82 g/L, which was 23.7% than the control group. As the Na_2_HPO_4_ concentration further increased, the 3-HB concentration decreased sharply. At the Na_2_HPO_4_ concentration of 24 mM, the production rate of 3-HB was still maintained at a high level, but the concentration of 3-HB at the end of the fermentation period was significantly lower even than that at 12 mM Na_2_HPO_4_. However, the concentration of 3-HB was significantly higher than the control group. The lowest 3-HB concentration of 13.52 g/L was obtained at 28 mM Na_2_HPO_4_, which was 11.2% lower than the control group.

The effects of different Na_2_HPO_4_ concentrations on PHB production are shown in Fig. 3b. With the increase in Na_2_HPO_4_ concentration from 0 to 16 mM, the PHB concentration increased at a relatively stable rate. The highest PHB concentration was obtained as 4.74 g/L, which was 47.7% higher than the control group. The PHB content also increased with the proper increase in Na_2_HPO_4_ concentration, and the highest PHB content was obtained at 12 mM Na_2_HPO_4_ as 64.3%, which was 21.8% higher than the control group. As the Na_2_HPO_4_ concentration further increased from 16 to 28 mM, both concentration and content of PHB decreased fast. When the Na_2_HPO_4_ concentration reached 20 mM, both concentration and content of PHB were significantly lower than the highest values but still significantly higher than the control group. The lowest PHB content was obtained at 28 mM Na_2_HPO_4_, which was 15.2% lower than the control group. However, the PHB concentration at 28 mM Na_2_HPO_4_ was not significantly different from the control group.

As shown in Fig. 3c, the LA concentration decreased as the Na_2_HPO_4_ concentration increased from 0 to 20 mM. The lowest LA concentration was obtained as 14.63 g/L, which was 31.9% lower than the control group. With the further increase in Na_2_HPO_4_ concentration from 20 to 28 mM, the LA concentration did not significantly change. No significant difference of AC concentration was observed at Na_2_HPO_4_ concentrations between 0 to 20 mM. However, the AC concentration increased significantly as the Na_2_HPO_4_ concentration further increased. The highest AC concentration was obtained at 28 mM as 8.08 g/L, which was 63.6% higher than the control group.

The fermentation performance of *B. cepacia* on the production of 3-HB and PHB is shown in Table 4. Both highest productivity and highest yield of 3-HB were obtained at 20 mM Na_2_HPO_4_, which were 36.7% and 23.7% higher than the control group. Both highest productivity and highest yield of PHB were obtained at 16 mM Na_2_HPO_4_, which were 54.9% and 47.9% higher than the control group.

**Table 4:** The productivity and yield of 3-HB and PHB at different Na_2_HPO_4_ concentrations

Concentration, mM	Productivity, g/(L × h)		Yield, g/g glucose	
	3-HB	PHB	3-HB	PHB
0	0.073 ± 0.002	0.015 ± 0.002	0.298 ± 0.005	0.063 ± 0.006
4	0.075 ± 0.002	0.018 ± 0.002	0.310 ± 0.006	0.073 ± 0.006
8	0.083 ± 0.002	0.020 ± 0.002	0.340 ± 0.006	0.083 ± 0.008
12	0.090 ± 0.002	0.023 ± 0.003	0.354 ± 0.005	0.091 ± 0.009
16	0.094 ± 0.002	0.024 ± 0.002	0.368 ± 0.005	0.093 ± 0.008
20	0.099 ± 0.001	0.022 ± 0.002	0.369 ± 0.004	0.081 ± 0.008
24	0.084 ± 0.002	0.017 ± 0.002	0.330 ± 0.005	0.067 ± 0.007
28	0.065 ± 0.002	0.014 ± 0.001	0.265 ± 0.006	0.056 ± 0.004

During the fermentation process, the Na_2_HPO_4_ can provide essential phosphorus for cell growth and metabolism, which is usually utilized as the important substrate for the synthesis of adenosine triphosphate (ATP) and nicotinamide adenine dinucleotide phosphate (NADPH) (Freches and Lemos 2017). At present, many studies have pointed out that the limitation of phosphorus has a positive effect on the synthesis and accumulation of PHB. However, the positive effect of proper Na_2_HPO_4_ concentration on PHB synthesis under the nitrogen source limitation has been reported (Moreno et al. 2015). It has been confirmed that NADPH is a key substance in the PHB synthesis process (Kessler and Witholt 2001). The increase in the concentration of NADPH will inhibit the activity of citrate synthase, thereby promoting the flow of acetyl-CoA to the PHB synthesis pathway instead of the TCA cycle. In the PHB synthesis pathway, the substrate produced by PHB is 3-hydroxybutyrate-CoA, which is generated by the conversion of acetoacetyl-CoA. The conversion of hydroxybutyrate-CoA to acetoacetyl-CoA requires the consumption of NADPH (Wang et al. 2019). Therefore, a high level of NADPH concentration can effectively promote the synthesis and accumulation of PHB. Appropriate addition of Na_2_HPO_4_ can promote the synthesis of NADPH by providing essential phosphorus, thereby improving the yield and production efficiency of PHB. In addition, the appropriate addition of Na_2_HPO_4_ will promote the synthesis of ATP, which improves the uptake efficiency of the carbon source, thereby increasing the efficiency of PHB synthesis (Lai and Lan 2021). The increase in 3-HB concentration and production efficiency is also due to the promotion of the overall cell metabolism caused by the increased level of ATP synthesis promotes, thereby simultaneously stimulating the decomposition of PHB. As the concentration of Na_2_HPO_4_ increased, a higher phosphorus concentration results in an increase in the size of the total ATP pool, which significantly promotes cell growth instead of PHB synthesis (García et al. 2021). The increase in cell growth activity promotes the decomposition of PHB, which leads to an increase in the concentration of 3-HB. Higher phosphorus concentrations will also lead to an increase in the level of intracellular NADP^+^ concentration, resulting in a lower PHB production efficiency due to a lower ratio of NADPH and NADP^+^ (García et al. 2018). Therefore, higher concentrations of Na_2_HPO_4_ have a negative effect on the accumulation of PHB. In addition, a higher concentration of phosphorus promotes the conversion of acetyl-CoA to acetyl-P as a substrate, and the acetyl-P can be further converted into acetate by the catalysis of acetate kinase (Park et al. 2019). It explains the reason for an increase in acetate concentration with the increase in Na_2_HPO_4_ concentration. The further increased phosphorus concentration leads to a higher cell growth activity and lower level of NADPH synthesis, which resulted in a further decrease in the production efficiency of PHB (Dalsasso et al. 2019). The decrease in PHB production resulted in a decrease in 3-HB concentration and production efficiency. The decrease in 3-HB concentration is also caused by more 3-HB consumption for acetyl-CoA regeneration to produce acetate.

### Effects of Na_***3***_C_**6**_H_**5**_O_***7***_on the production of 3-HB, PHB, and by-products

**Fig. 4:**
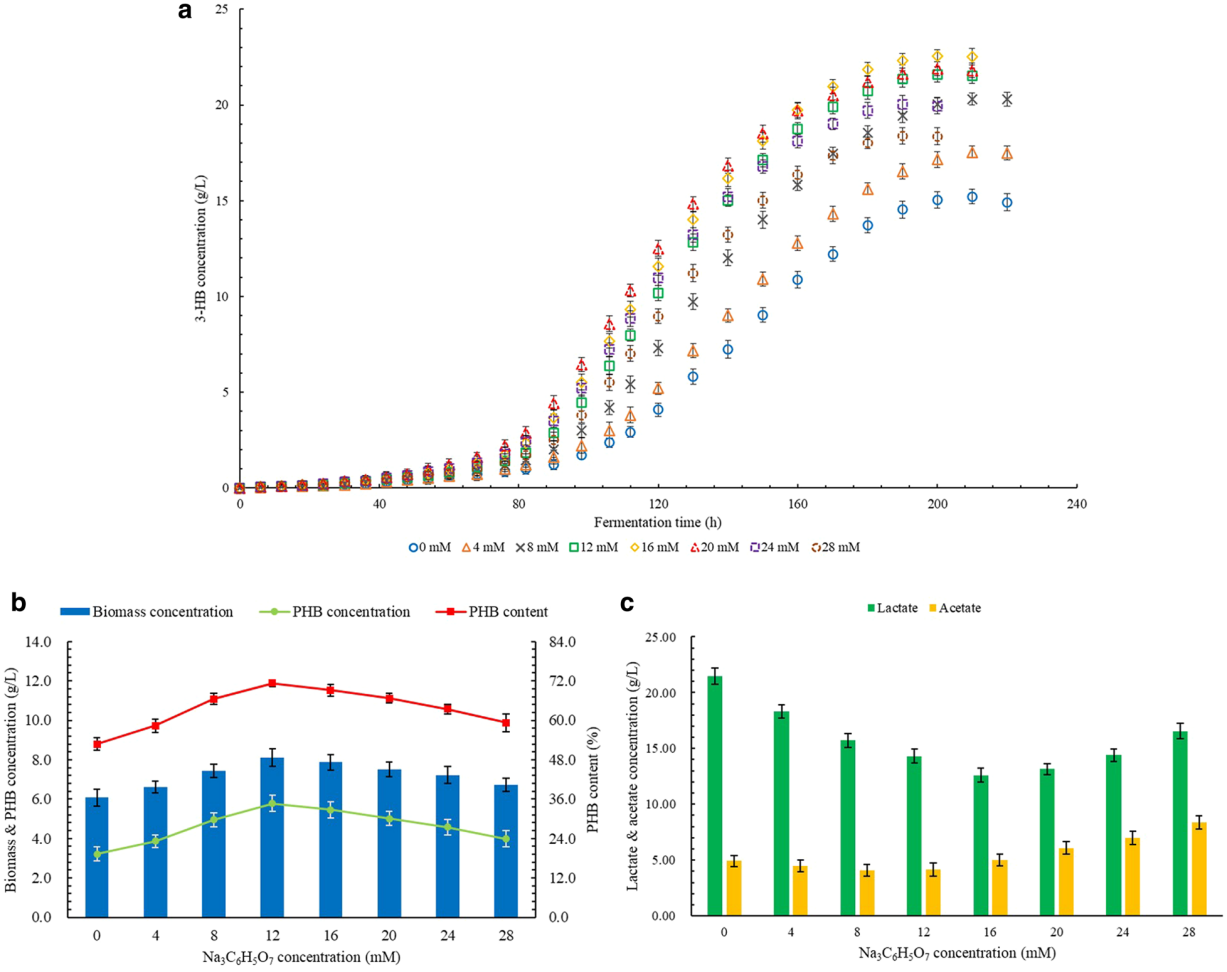
**a** Effects of Na3C6H5O7 on the production of 3-HB. **b** Effects of Na3C6H5O7 on the production of PHB. **c** Effects of Na3C6H5O7 on the production of LA and AC

The effect of different Na_3_C_6_H_5_O_7_ concentrations on the production of 3-HB is shown in Fig. 4a. The proper increase in Na_3_C_6_H_5_O_7_ concentration led to an increase in 3-HB production. The highest 3-HB concentration of 22.56 g/L was obtained at 16 mM Na_3_C_6_H_5_O_7_, which was 48.2% higher than the control group. When the Na_3_C_6_H_5_O_7_ concentration increased to 20 mM, the 3-HB concentration at the end of the fermentation period was lower than that at 16 mM, but the production rate of 3-HB in a certain period was higher than that at 20 mM Na_3_C_6_H_5_O_7_. As the Na_3_C_6_H_5_O_7_ concentration increased to 24 mM, the production rate of 3-HB at the middle stage of the fermentation period was still maintained at a high level, but the final concentration of 3-HB significantly decreased. The production rate and concentration of 3-HB at 28 mM Na_3_C_6_H_5_O_7_ further decreased, but the 3-HB concentration of 18.37 g/L was still 20.7% higher than the control group.

The results of PHB production at different Na_3_C_6_H_5_O_7_ concentrations are shown in Fig. 4b. With the increase in Na_3_C_6_H_5_O_7_ concentration, the concentration and content of PHB increased significantly. The highest concentration and content of PHB were obtained at 12 mM Na_3_C_6_H_5_O_7_ as 5.78 g/L and 71.3%, respectively, which were 80.1% and 35.0% higher than the control group. As the Na_3_C_6_H_5_O_7_ concentration further increased, the concentration and content of PHB decreased slowly. The concentration and content of PHB at 28 mM Na_3_C_6_H_5_O_7_ were 3.98 g/L and 59.3%, respectively, which were still 24.0% and 12.3% higher than the control group.

The production of by-products affected by different levels of Na_3_C_6_H_5_O_7_ concentration is shown in Fig. 4c. As the Na_3_C_6_H_5_O_7_ concentration increased properly, the concentrations of LA and AC decreased significantly. The lowest LA concentration of 12.61 g/L was obtained at 16 mM Na_3_C_6_H_5_O_7_, which was 41.3% lower than the control group. The lowest AC concentration was obtained at 8 mM Na_3_C_6_H_5_O_7_ as 4.07 g/L, which was 17.6% lower than the control group. The further increase in Na_3_C_6_H_5_O_7_ concentration resulted in increased concentrations of LA and AC. At the Na_3_C_6_H_5_O_7_ concentration of 28 mM, the LA concentration was 16.56 g/L, which was still 22.9% lower than the control group. However, the AC concentration at 28 mM Na_3_C_6_H_5_O_7_ was 8.36 g/L, which was 69.6% higher than the control group.

Table 5 shows the productivity and yield of 3-HB and PHB affected by different levels on Na_3_C_6_H_5_O_7_ concentration. Both highest productivity and highest yield of 3-HB were obtained at 16 mM Na_3_C_6_H_5_O_7_, which were 55.6% and 48.3% higher than the control group. Both highest productivity and highest yield of PHB were obtained at 12 mM Na_3_C_6_H_5_O_7_, which were 47.1% and 80.0% higher than the control group. The productivity and yield of 3-HB and PHB at 28 mM were all higher than the control group.

**Table 5:** The productivity and yield of 3-HB and PHB at different Na_3_C_6_H_5_O_7_ concentrations

Concentration, mM	Productivity, g/(L × h)		Yield, g/g glucose	
	3-HB	PHB	3-HB	PHB
0	0.073 ± 0.002	0.015 ± 0.002	0.298 ± 0.005	0.063 ± 0.006
4	0.084 ± 0.002	0.018 ± 0.001	0.344 ± 0.004	0.076 ± 0.006
8	0.097 ± 0.002	0.024 ± 0.002	0.398 ± 0.004	0.097 ± 0.006
12	0.108 ± 0.002	0.029 ± 0.002	0.423 ± 0.004	0.113 ± 0.007
16	0.113 ± 0.002	0.027 ± 0.002	0.445 ± 0.004	0.107 ± 0.007
20	0.110 ± 0.002	0.025 ± 0.002	0.429 ± 0.004	0.098 ± 0.006
24	0.106 ± 0.003	0.024 ± 0.002	0.393 ± 0.005	0.090 ± 0.007
28	0.097 ± 0.002	0.021 ± 0.002	0.360 ± 0.005	0.078 ± 0.007

Citrate is an important intermediate in the TCA cycle, which is generated from acetyl-CoA by the catalysis of citrate synthase. Na_3_C_6_H_5_O_7_ can provide C_6_H_5_O_7_^3−^ to participate in cell metabolism, thereby regulating cell growth and metabolite synthesis. At present, the positive effect of Na_3_C_6_H_5_O_7_ on PHB synthesis has been reported, but its related research is still limited (Samantaray and Mallick 2012). The addition of citrate can effectively inhibit the activity of citrate synthase. Therefore, more acetyl-CoA flows into the PHB synthesis pathway to participate in the production of PHB and 3-HB. It may be the main reason for the improvement of PHB and 3-HB production at a suitable Na_3_C_6_H_5_O_7_ concentration. In addition, studies have reported the inhibitory effect of Na_3_C_6_H_5_O_7_ on the Embden–Meyerhof–Parnas (EMP) pathway, which is resulted from the decrease in phosphofructokinase activity caused by the chelation of Na_3_C_6_H_5_O_7_ and magnesium ions (Chen et al. 2010). It leads to more glucose flowing into the pentose phosphate pathway in the form of glucose-6-phosphate, thereby promoting the synthesis of NADPH. The increase in the production level of NADPH also promotes the synthesis and accumulation of PHB. The inhibition of the EMP pathway leads to a decrease in the overall pyruvate synthesis level, but the increase in the NADPH synthesis level promotes the conversion of pyruvate into acetyl-CoA and further flows into the PHB synthesis pathway, which leads to a reduction in the pyruvate flowing into the LA synthesis pathway. This may explain the decrease in LA concentration with the appropriate increase in the Na_3_C_6_H_5_O_7_ concentration. A higher concentration of Na_3_C_6_H_5_O_7_ caused a significant increase in the concentration of LA and AC and a decrease in the concentration of PHB. However, within a certain range of Na_3_C_6_H_5_O_7_ concentration, the degradation of PHB was promoted, which led to a rapid increase in the concentration of 3-HB. This may be because sodium citrate not only directly affects the activity of specific enzymes in multiple metabolic pathways, but also indirectly affects the overall efficiency of cell growth and metabolism by changing the osmotic pressure of the fermentation environment (Samantaray and Mallick 2012). The specific mechanism needs to be further studied. However, citrate can be used as a carbon source to participate in cell metabolism, which improves the ratio of carbon and nitrogen in the fermentation medium, thereby weakening the negative effects of high concentrations of sodium citrate on the production of 3-HB and PHB to a certain extent.

### Effects of nitrogen sources on the production of 3-HB, PHB, and by-products

**Fig. 5:**
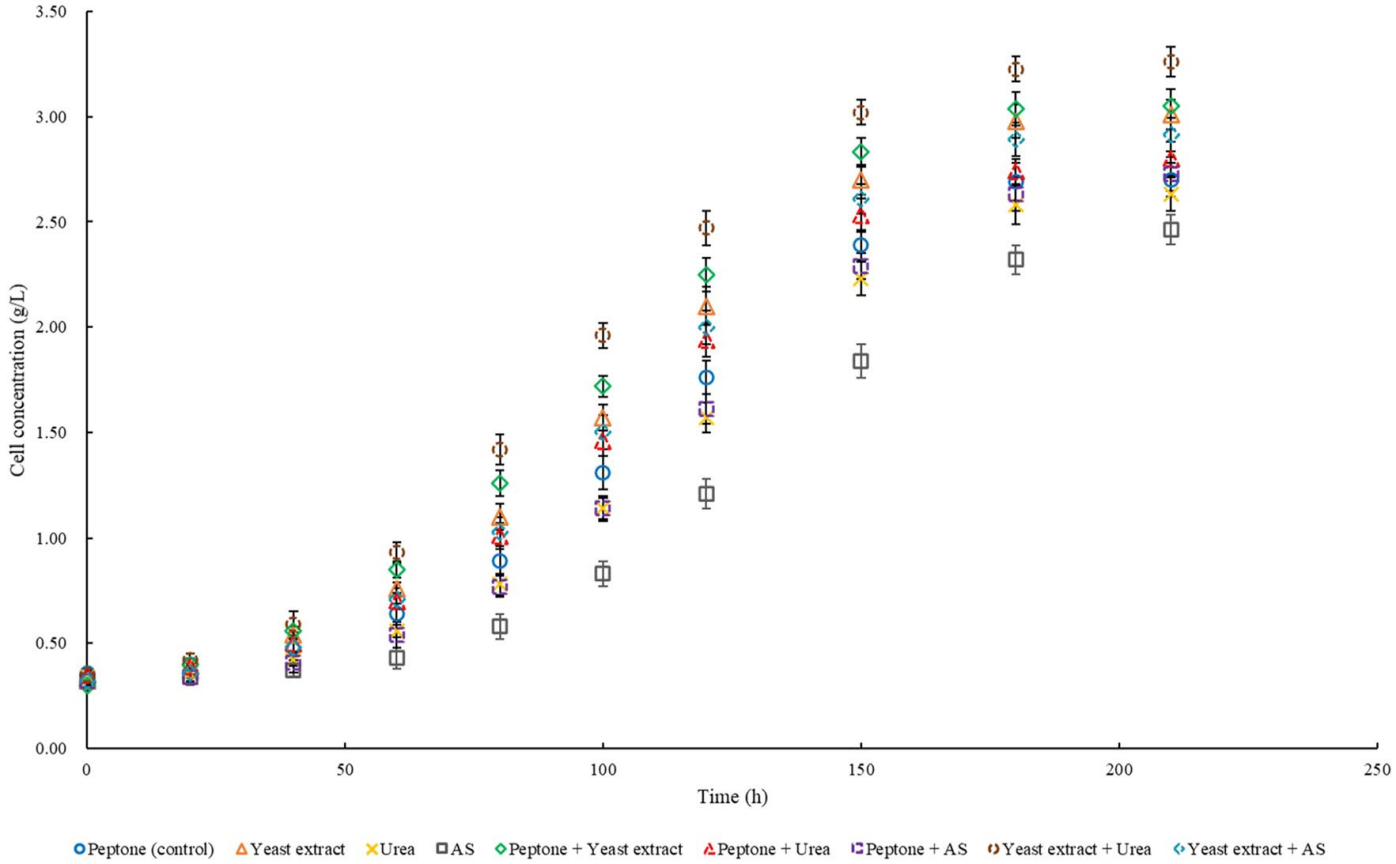
Effects of different nitrogen sources on net cell concentration

The production of various products affected by different types of nitrogen sources is shown in Table 6. Compared to that under the condition of the single nitrogen sources, a higher production capacity of 3-HB and PHB can be obtained under the condition of the mixed nitrogen sources. In addition, the production of the two by-products is more effectively reduced with the addition of mixed nitrogen sources. The highest production capacity of 3-HB was obtained under the mixed nitrogen source of yeast extract and ammonium sulfate (AS), while the highest production capacity of PHB was obtained under the mixed nitrogen source of yeast extract and urea. Both lowest production capacities of 3-HB and PHB were obtained under the single nitrogen source of AS. The net cell growth using different nitrogen sources is shown in Fig. 5. Compared to the medium without yeast extract, a higher net cell concentration and a shorter lag phase can be observed in the medium containing yeast extract. The highest activity of cell growth was also observed when yeast extract was combined with urea. It indicated that a proper increase in cell concentration effectively enhance the production of both PHB and 3-HB.

**Table 6:** The production of 3-HB, PHB, and by-products under the condition of different nitrogen sources

Type	3-HB concentration, g/L	3-HB productivity, g/(L × h)	3-HB yield, g/g glucose	PHB concentration, g/L	PHB content, %	PHB productivity, g/(L × h)	PHB yield, g/g glucose	LA concentration, g/L	AC concentration, g/L
Peptone (control)	15.22 ± 0.38	0.073 ± 0.002	0.298 ± 0.005	3.21 ± 0.35	54.4 ± 1.9	0.015 ± 0.002	0.063 ± 0.006	21.48 ± 0.74	4.94 ± 0.49
Yeast extract	15.89 ± 0.53	0.076 ± 0.003	0.312 ± 0.007	3.67 ± 0.29	55.2 ± 1.7	0.018 ± 0.002	0.074 ± 0.007	21.76 ± 0.68	5.42 ± 0.56
Urea	15.57 ± 0.57	0.074 ± 0.003	0.305 ± 0.008	3.04 ± 0.32	53.6 ± 2.3	0.015 ± 0.002	0.061 ± 0.006	21.08 ± 0.81	5.01 ± 0.42
AS	14.63 ± 0.47	0.070 ± 0.002	0.287 ± 0.007	2.30 ± 0.36	48.3 ± 2.1	0.011 ± 0.002	0.045 ± 0.007	20.53 ± 0.82	4.90 ± 0.47
Peptone + Yeast extract	16.35 ± 0.53	0.078 ± 0.003	0.321 ± 0.007	3.79 ± 0.39	55.5 ± 2.1	0.017 ± 0.002	0.070 ± 0.008	18.91 ± 0.65	4.86 ± 0.51
Peptone + urea	16.91 ± 0.55	0.081 ± 0.003	0.332 ± 0.007	3.85 ± 0.41	57.9 ± 2.5	0.018 ± 0.002	0.075 ± 0.008	19.32 ± 0.71	4.03 ± 0.58
Peptone + AS	17.02 ± 0.39	0.081 ± 0.00s2	0.334 ± 0.005	3.53 ± 0.39	56.4 ± 1.7	0.017 ± 0.002	0.069 ± 0.007	20.12 ± 0.80	4.45 ± 0.39
Yeast extract + urea	17.16 ± 0.42	0.082 ± 0.002	0.337 ± 0.005	4.68 ± 0.30	59.2 ± 2.4	0.022 ± 0.002	0.091 ± 0.006	18.36 ± 0.87	3.87 ± 0.45
Yeast extract + AS	17.34 ± 0.47	0.083 ± 0.002	0.340 ± 0.006	3.74 ± 0.38	56.2 ± 2.8	0.018 ± 0.002	0.073 ± 0.007	19.51 ± 0.79	4.23 ± 0.53

Compared to other single nitrogen sources, the highest production capacity of PHB and 3-HB was obtained when yeast extract was used. As a complex nitrogen source, yeast extract contain various nutrients and promoters for cell growth and metabolism, which might have a positive effect on 3-HB and PHB production (Sharma et al. 2017). As another type of complex nitrogen source, peptone can still maintain the production capacity of 3-HB and PHB at a relatively high level, but its effect is weaker than that of yeast extract. It might be because the composition in yeast extract is more easily taken up and utilized by *B. cepacia* cells. Urea is a type of organic nitrogen source with a smaller molecular size and stronger polarity, which can be efficiently used by cells (Dañez et al. 2020). Therefore, it can be also regarded as a suitable single nitrogen source for 3-HB and PHB production. As a type of inorganic nitrogen source, AS has been widely used for PHB production (El-Nahrawy et al. 2018). However, since AS exists in the form of ions carrying charges in the fermentation medium, it has a relatively lower efficiency of uptake and utilization by cells compared to organic nitrogen sources. It might explain the reason for a lower production capacity of PHB and 3-HB when using AS as the nitrogen source in this study. At present, studies have reported the positive effects of mixed nitrogen sources on the synthesis of PHB (Sharma et al. 2017). Therefore, the choice of nitrogen source can be modified based on specific strains, substrates, and fermentation conditions. An appropriate increase in the proportion of inorganic nitrogen sources in the mixed nitrogen source can increase the synthesis and decomposition efficiency of PHB at the same time, thereby simultaneously increasing the production capacity of PHB and 3-HB (Wei et al. 2011).

## Conclusion

Various sodium salts have a positive effect on the production of 3-HB and PHB at appropriate concentrations. Among them, the appropriate addition of Na_3_C_6_H_5_O_7_ can significantly promote the production of 3-HB and PHB and reduce the synthesis of by-products, thereby effectively improving the conversion rate of carbon sources and the production efficiency of products. Sodium chloride is not conducive to the improvement of fermentation efficiency. Compared to a single nitrogen source, a mixed nitrogen source has greater advantages to produce 3-HB and PHB. This study focused on the effects of sodium salt-based anions and nitrogen sources as nutrients on the production of 3-HB and PHB. The results of this study can provide relevant support for the selection and further standardization of medium components for the production of 3-HB and PHB.

## Data Availability

The datasets used and/or analyzed during the current study are available from the corresponding author on reasonable request.
